# Effects of BOPPPS combined with TBL in surgical nursing for nursing undergraduates: a mixed-method study

**DOI:** 10.1186/s12912-023-01281-1

**Published:** 2023-04-23

**Authors:** Zhiying Li, Xiaoyan Cai, Kebing Zhou, Jieying Qin, Jiahui Zhang, Qiaohong Yang, Fengxia Yan

**Affiliations:** School of Nursing, Jinan University, 601 Huangpu Avenue West, Tianhe District, 510632 Guangzhou, China

**Keywords:** Surgical nursing, BOPPPS, TBL, Autonomous learning competencies, Critical thinking

## Abstract

**Background:**

Surgical Nursing is a core subject for nursing undergraduates that requires active and effective learning strategies to cultivate students’ autonomous learning competencies and critical thinking. The effects of BOPPPS (Bridge-in, Objectives, Pretest, Participatory Learning, Post-test and Summary) model combined with team-based learning (TBL) have rarely been explored in Surgical Nursing courses.

**Objective:**

To explore the effects of BOPPPS combined with TBL in Surgical Nursing for nursing undergraduates.

**Methods:**

A mixed research method of quasi-experimental study design and descriptive qualitative research was used. The control group included 27 nursing undergraduates who had finished the Surgical Nursing course using traditional learning. The experimental group included 36 nursing undergraduates were enrolled in to receive the Surgical Nursing course in the teaching mode of BOPPPS combined with TBL. The quantitative data of students’ Surgical Nursing final scores, autonomous learning competencies and critical thinking ability of the two groups were collected and compared by *t*-test. Qualitative results were obtained through semi-structured interviews and data were analyzed by thematic analysis method.

**Results:**

Compared with the traditional learning mode, BOPPPS combined with TBL significantly improved nursing students’ final examination scores, autonomous learning competencies and critical thinking ability (*p* < 0.05). Qualitative results from 14 undergraduate nursing students’ interviews were summarized into five themes: (1) stimulating learning interest; (2) improving autonomous learning ability; (3) improving the sense of teamwork; (4) exercising critical thinking; and (5) suggestions for improvement.

**Conclusions:**

The combination of BOPPPS and TBL positively impacted nursing students by improving their autonomous learning competencies and critical thinking ability. The study suggests BOPPPS combined with TBL learning as an effective, alternative learning mode.

**Supplementary Information:**

The online version contains supplementary material available at 10.1186/s12912-023-01281-1.

## Introduction

In nursing education, Surgical Nursing is a core subject for nursing undergraduates, which combines the natural science attribute of surgery with the humanistic care connotation of nursing. It attaches equal importance to nursing theory and nursing practice, aiming to cultivate high-quality nursing talents. Notably, the cultivation of nursing talents focuses on not only professional nursing knowledge, but also nursing abilities, including autonomous learning competencies, critical thinking ability, and clinical skills [1–3]. Correspondingly, clinical nursing should have been fully equipped with adequate competencies to carry out peri-operative nursing since they were students [4, 5].

Surgical Nursing course, covering a wide range of knowledge, was usually delivered through traditional lecture teaching in the past. However, the traditional lecture teaching, mainly featured by teacher output and students’ passive input, may result in a gap in theoretical learning and clinical practice [6]. Additionally, lacking interaction in traditional teaching might be not conducive to cultivate students’ teamwork ability, autonomous learning ability and divergent thinking [7] and therefore will add the difficulties of engaging in clinical surgical nursing practice to nursing students [8, 9]. Therefore, new teaching mode is urgently needed to deliver Surgical Nursing course, so as to efficiently improve students’ autonomous learning competencies and critical thinking ability.

The model of Bridge-in, Objectives, Pretest, Participatory Learning, Post-test and Summary, BOPPPS for short, is a student-centered and closed-loop teaching model. This teaching model, based on constructivism and communicative approach, emphasizes students’ participatory interaction and feedback [10, 11]. It can provide more operational ideas and methods for teaching design, help to attract students to participate in the class, and thus achieve better teaching results [12]. Recently, this model has been applied to medical education, and exerts satisfactory teaching effect [13]. The BOPPPS-based teaching could stimulate students’ interest and enthusiasm, and improve their thinking ability [10, 14] and academic performance and learning initiative [10, 11, 15]. The BOPPPS model, as an open instructional design model, can be incorporated with diverse teaching methods to be more in line with medical students’ psychological characteristics and cognitive law [10].

Another learner-centered instructional strategy, team-based learning (TBL) refers to divide students into several small groups, in which students solve problems through mutual learning and cooperation within a group [16, 17]. Team-based learning is a group active learning process, and helps to deepen students’ impression of knowledge through individual test, team test, and team practice and application [18]. Besides, TBL emphasizes students’ teamwork, in which all members systematically complete learning activities under a well-organized curriculum design. It can improve students’ ability of communication, teamwork, problem-solving and critical thinking by promoting active learning and student participation [19].

Both BOPPPS and TBL have been applied in the teaching of medical specialty, with good teaching feedback [10, 18, 20]. However, the combination of BOPPPS with TBL has rarely been applied in the Surgical Nursing courses. In this study, we explored the teaching effects of integrating TBL into the BOPPPS model in Surgical Nursing course, aiming to investigate the effects of this new teaching mode on nursing undergraduates’ learning outcomes, including students’ knowledge mastery, autonomous learning competencies and critical thinking.

## Materials and methods

### Design

A mixed research method of quasi-experimental study design and descriptive qualitative research was used to set up a control group and an experimental group.

### Participants and setting

A total of 63 nursing undergraduates from two different grades were included in this study by means of convenient sampling. The inclusion criteria were: (1) full-time nursing undergraduate students; (2) no Surgical Nursing learning experience of TBL and BOPPPS; (3) no current physical or psychiatric symptoms; (4) voluntarily participate in this study and sign the informed consent form. The control group included 27 students and received the traditional teaching model, while 36 students were enrolled in the experimental group and received the new teaching model of BOPPPS combined with TBL.

All students were directly recruited into university after passing the national university entrance exam in high school, with a total of 12 years of education. They all received the Surgical Nursing courses in the first semester of the third year at university, and had previously followed the same nursing curricula (including relevant basic curricula, nursing basic, surgical nursing, internal medicine nursing) in the first two years. Two groups of students used the same version of the Surgical Nursing textbook, and were taught by the same instructors. Additionally, the characteristics of the two groups of students (including age, gender, and Grade Point Average before the course), autonomous learning competencies and critical thinking were evaluated before the experiment, and the results indicated that the two groups were comparable. Data were also collected at the end of the courses, in December 2020 and December 2021.

### Educational intervention

#### Teaching method of control group

Traditional teaching methods were used in the control group. Before the class, the teacher announced the content of the next course, and the students were free to preview. In class, the teacher spoke and the students listened.

#### Teaching method of experimental group

##### Setting up a teaching team

Surgical Nursing teaching team was composed of teachers with more than five years of teaching experience and rich nursing practice experience. They were trained with the theory and teaching skills of BOPPPS and TBL.

##### Implementing the educational reform plan

The BOPPPS combined with TBL instructional design was divided into six steps, which were carried out before, during and after the class.

##### (1) Establish a mixed learning team

 Reasonable grouping is the precondition of cooperative learning, and ‘homogeneity between groups, heterogeneity within groups’ is the basic principle of grouping for cooperative learning [21]. To strengthen group learning and achieve optimal learning outcomes, each group had a maximum of six students. Therefore, before class, the teacher determined the groups according to the students’ average grade point, so that the overall level of learning ability of each group was equal. Eventually, students in the intervention group were divided into six groups. Adjustment of group members took place in the first week based on teachers’ feedback on student performance. Within each group, one student was selected as group leader, responsible for organizing group activities. Group composition remained fixed throughout the course. The clear assignment of teamwork ensured the participation of each member and promoted the collaboration within the groups.

##### (2) Pre-class preparation

The teacher assigned pre-assessment tasks according to teaching objectives, and sent learning courseware and online micro-videos to students through the WeChat group. Each group leader organized the pre-course preview, supervised the group members to complete the individual readiness assessment test (iRAT), and collected the difficulties encountered in the preview and submitted them to the teacher the day before class.

##### (3) Classroom teaching

The BOPPPS instructional design in the classroom is mainly divided into the following six sections.

##### Bridge-in (B)

This section focuses on explaining the importance of the course and arousing students’ interest. Given the characteristics of the Surgical Nursing course is diversified, divergent and practical, the course theme was introduced by using diverse cases.

##### Objectives (O)

The teaching objectives should be clear and explicit, which can comprehensively demonstrate the three-dimensional objectives of knowledge and skills, process and methods, and emotional attitude and values. Since Surgical Nursing is characterized by a large amount of professional knowledge, students’ theoretical basis is highly emphasized, which includes medical knowledge related to diseases as well as specialized nursing procedures and theories. The process and method focus on the practical application of nursing procedures. For example, in response to the given cases, students were able to perform nursing assessment and nursing diagnosis, develop and implement a nursing plan for the patient. The goal of emotional attitude and values is to cultivate students’ professional quality and humanistic care ability in Surgical Nursing. Therefore, we present the learning objectives to the students before class and emphasized them again in class.

##### Pretest (P)

The test before lesson can reveal the level of students’ learning basis and ability. Pre-course tests focus on basic medical knowledge about diseases in Surgical Nursing. Then, teacher explained the key and difficult knowledge points in a targeted manner according to students’ responses, and clarified their ambiguous knowledge points during class. Pretest may lead to effective previews, and therefore improve the learning efficiency in the classroom.

##### Participatory learning (P)

Students is the main body of this part. Multiple ways were used to achieve teacher-student interaction, which is the core section of BOPPPS combined with TBL teaching. First of all, a case analysis question was published through the “Rain Classroom” APP, and the team readiness assessment test (tRAT) was conducted, and the answer was submitted when all team members reached a consensus after discussion. Then, according to class progress scheduling, scenarios simulation, classroom demonstration and other methods were selected to strengthen students’ application of knowledge. Participatory learning is the integration of process and goal, and thus is the important section of teaching process. Teachers were required to flexibly use teaching media and resources to create a relaxed learning environment for students and stimulated students’ interest in participating in the learning.

##### Post-test (P)

At the end of the class, teacher evaluated the students’ knowledge mastery according to the course objectives. The test forms (including oral questions, online exercise questionnaires, group assignments after class, etc.) were selected by the teacher flexibly according to the teaching contents. In addition, the evaluation of teaching effect is also one of the purposes of this section, including students’ feedback score to each teammate and students’ evaluation on this class, which provides a reference for optimizing teaching design and teaching process.

##### Summary (S)

Finally, teacher led students to summarize the knowledge of the course, and helped them to establish a complete knowledge framework. At the same time, students were also encouraged to orally make brief summaries, so as to strengthen their memorization of the course contents.

##### (4) After-class tutoring

**Fig. 1:**
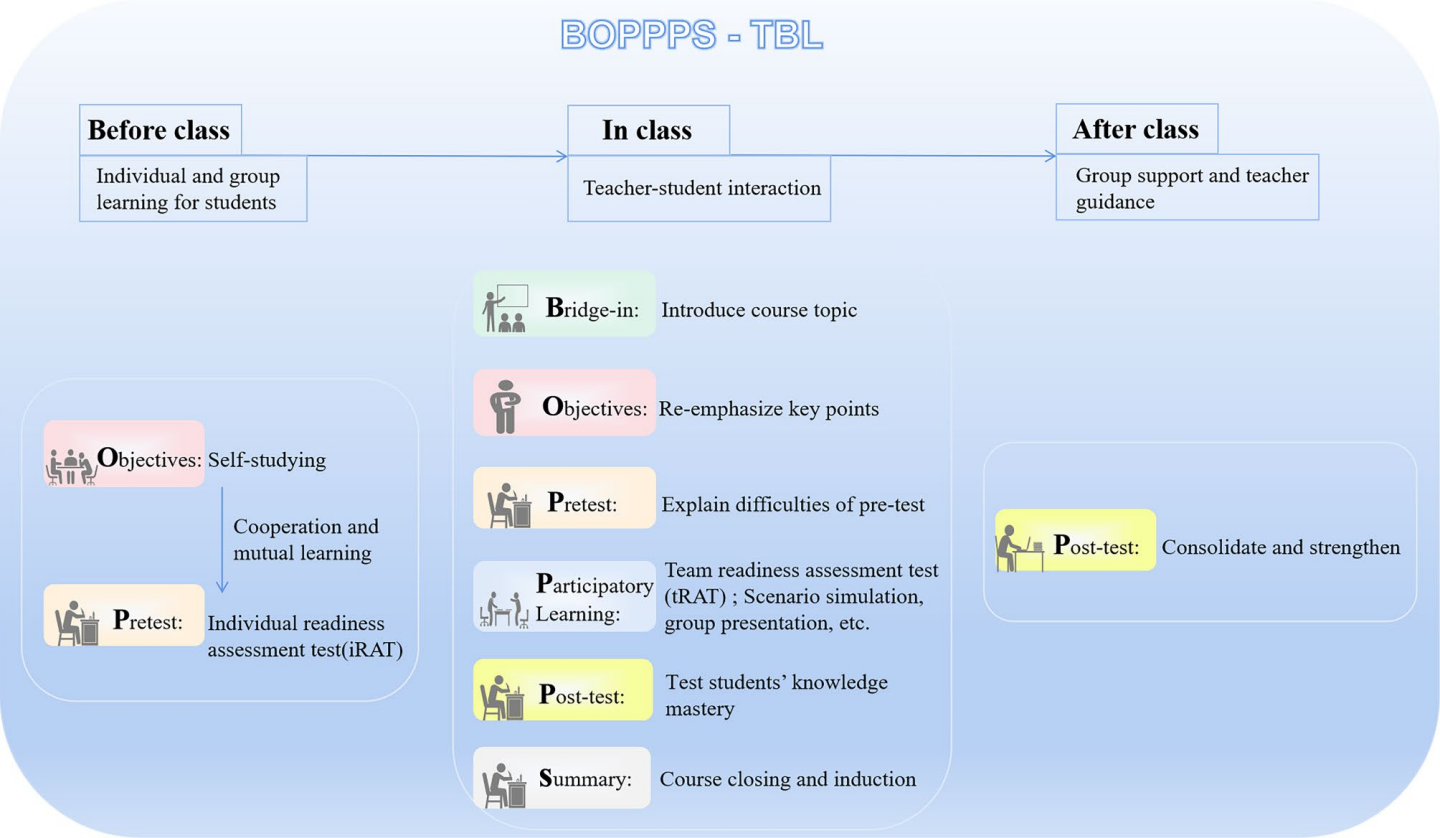
Flow chart of BOPPPS-TBL instructional design

In order to help students fully consolidate the content learned in the classroom, teachers offered practice materials to students through the WeChat group after class. If students encountered difficulties and were unable to complete the task on their own, they could carry out group discussions and make joint efforts to figure out the solutions. When problems could not be solved through team efforts, teachers offered the ways to address the problems rather than directly provided answers (see Fig. 1; Table 1 for details).

**Table 1:** The chapter on Colorectal Cancer is used as an example to show the specific teaching process of BOPPPS combined with TBL

BOPPPS	Purpose	Content of courses
Bridge-in	Introduce the subject of course by using cases, and current events and the latest data were used to stimulate students’ interest.	Mr. Li, 62 years old, was admitted to the hospital because of a change in defecation habit for 2 months and blood in the stool for 1 month. Diagnosis: low rectal cancer. After admission, low anterior rectum resection was performed under general anesthesia. On the 3rd day after operation, the patient was fed with liquid diet after fart and had sudden abdominal pain on the 5th day after the operation. Physical examination: T 37.8℃, P 98/min, R 20/min, BP 130/80mmHg, palpation of abdomen showed obvious signs of peritonitis, increased drainage volume, and turbid fluid in the drainage tube.
Objectives	Show three-dimensional teaching objectives to help students define their learning tasks. ①Before class: guide students to study independently according to the learning objectives, and encourage them to have group mutual discussion. ②In class: emphasize learning objectives again to arouse students’ attention.	①Knowledge objective: master the clinical manifestations and management principles of colorectal cancer. ②Ability goal: the use of nursing procedures for colorectal cancer patients to implement holistic nursing. ③Quality objective: form the attitude and behavior of caring about the psychology of patients with colorectal cancer and respecting the privacy of patients with enterostomy.
Pretest	Check the effects of students’ preview. ①Before class: students are encouraged to complete the individual readiness assessment test after preview. ②In class: explain the key and difficult points in a targeted way to students.	①Basic medical knowledge: classification and staging of colorectal cancer, clinical manifestations and surgical methods. ②Basic knowledge of nursing: master the the information should be collected when assessing the patient’s physical condition. ③Nursing skills: master the ability to guide patients to make preoperative bowel preparation, provide postoperative care after enterostomy and catheter indwelling
Participatory learning	The team readiness assessment test is completed by case analysis, and standardized patients, scenario simulation, evidence-based nursing teaching and other methods are used to promote the interaction between teachers and students and improve students, so as to improve students’ participation in the course.	Firstly, taking nursing procedure as the framework, the nursing care of colorectal cancer patients was divided into five tasks: correctly evaluating the condition, putting forward the main nursing diagnosis, making personalized nursing plan, implementing nursing and evaluating nursing effect.
Post-test	Have a comprehensive understanding of students’ knowledge mastery and teachers’ teaching effect.	The time-limited test questionnaire was distributed through WeChat group, and the question type and content were referenced nurse practice examination. After answering the questions, students were encouraged to analyze them first, and then teachers made comments.
Summary	Help students sort out important and difficult points to further consolidate the learning content.	①Teacher summarized evaluated conditions, nursing diagnosis and nursing measures of colorectal cancer patients. ②Students were encouraged to use mind mapping to sort out the knowledge and skills they had learned to form a standardized nursing procedure for patients with colorectal cancer.

### Effectiveness assessment

At the end of the course, quantitative data were collected using the final theoretical examination score of Surgical Nursing, Autonomous Learning Competencies Scale and Critical Thinking Disposition Inventory of Chinese Version. Additionally, semi-structured interviews were used to collect qualitative data as a supplement to the teaching effect evaluation.

#### Measurement of students’ mastery of knowledge

The final theoretical examination of Surgical Nursing at the end of the semester was used to evaluate students’ knowledge mastery. To fully understand the students’ grasp of knowledge points, the examination mainly included the clinical manifestations of common surgical diseases, observation, nursing diagnosis, perioperative special nursing points, and prevention and emergency treatment of common complications. The questions were set by the teachers in the research group. Since the two groups took final examinations in different years, the specific questions of the two final exams were not exactly the same. In order to ensure the equal difficulty levels of the two final exams, the exams were well-designed to consist of 3 parts (i.e. basic knowledge, comprehension and comprehensive application accounting for 30%, 30% and 40%, respectively). The test duration was 90 min and the maximum total score was 100 points.

#### Measurement of students’ autonomous learning competencies

The Autonomous Learning Competencies Scale of nursing students, compiled by Chinese researchers on the background of Chinese culture [22], was used to evaluate students’ self-directed learning ability in our study. The scale comprises 28 items in three dimensions, including ten items on self-management ability, eleven items on information management ability and seven items on learning cooperation ability. A 5-point Likert scale was adopted, with 1 indicating ‘completely inconsistent’ and 5 indicating ‘completely consistent’, and a total score of 28–140 points. The total score is directly proportional to the level of independent learning ability, that is, higher scores indicate better independent learning ability. The Cronbach’s α coefficient of this scale was 0.86, indicated high internal consistency.

#### Measurement of students’ critical thinking

The Critical Thinking Disposition Inventory of Chinese Version (CTDI-CV), revised by Chinese nursing education experts [23], was used to evaluate nursing students’ critical thinking ability. The scale has seven dimensions: truth-seeking, open-mindedness, analytical ability, systematic ability, self-confidence in critical thinking, inquisitiveness and cognitive maturity. Each dimension contains ten items with a total of 70 items. A 6-point Likert scale was adopted, where 1 indicates ‘strongly disagree’ and 6 indicates ‘strongly agree’. Thirty positive items were positively assigned from 1 to 6, while forty negative items were inversely assigned. A score of one dimension ≥ 40 points indicates strong performance in a particular trait, and a total score ≥ 280 indicates positive critical thinking ability. The Cronbach’s α coefficient and content validity index of CTDI-CV were 0.90 and 0.89, respectively.

#### Qualitative evaluation-semi-structured interview

At the end of the course, a semi-structured interview was conducted to investigate the evaluation of undergraduate nursing students in the experimental group on the application of BOPPPS combined TBL model in Surgical Nursing. When selecting interviewees, we took students’ gender, age and scores into account, and then conducted purpose sampling in the experimental group to ensure the diversity of views. Students would not be included in interview when no new topics or information emerged in the interview.

In order to fully understand the teaching effect and students’ real experience of BOPPPS combined with TBL, the research team first conducted a preliminary interview with two students and determined the final interview outline: (1) How do you feel about BOPPPS combined with TBL learning in Surgical Nursing? (2) Do you think your study situation has changed since before? (3) What are the suggestions for BOPPPS combined with TBL teaching in Surgical Nursing?

A researcher, proficient in interview skills, was assigned to independently carry out the interviews. The interviews were conducted in a quiet and relaxed environment within 1 week after the course in order to avoid as many recall biases as possible. And each interview lasted about 20 min. The students’ conversations were recorded, but they were promised to keep the conversation confidential. The audio recordings of interview were transcribed verbatim into text within 24 h of the interview.

### Data analysis

Quantitative data of exam scores, autonomous learning competencies and critical thinking ability were described by mean values and standard deviation. The paired-sample t-tests were applied to compare the differences between pre-test and post-test, and the independent-sample t-tests were used to compare the differences between two groups. The Cohen’s d was calculated from the arithmetic means of the group, as well as the pooled standard deviation, to determine the effect size after implementing the teaching method. Cohen’s d of small, medium, and large effect size were defined as 0.2, 0.5, and 0.8, respectively [24]. Qualitative data obtained from semi-structured interviews were strictly analyzed by thematic analysis [25], which consisted of six steps: familiarizing the data, coding, generating themes, reviewing themes, defining and naming themes, and writing up.

## Results

### Characteristics of participants

The experimental group consisted of 36 students aged 18–24 (21.44 ± 1.443) years old, including 11 males and 25 females. Their Grade Point Average before the course was (3.23 ± 0.471). There were 27 students in the control group, aged 19–23 (21.30 ± 1.171) years, including 6 males and 21 females. Their Grade Point Average before the course was (3.14 ± 0.521). No significant differences in demographic characteristics were found between the two groups (*p* > 0.05) (see Table 2 for details).

**Table 2:** Comparison of demographic characteristics between the experimental and control groups

Characteristics		Experimental group (*n* = 36)	Control group (*n* = 27)	*p*-value
Age		21.44 ± 1.443	21.30 ± 1.171	0.664^b^
Gender				0.571^a^
	male	11 (30.6%)	6 (22.2%)	
	female	25 (69.4%)	21 (77.8%)	
Grade Point Average before the course		3.23 ± 0.471	3.14 ± 0.521	0.480^b^

Note: a: use independent-samples *t*-test to compare the gender in general; b: paired-sample *t*-test.

### Final examination results of the experimental group and the control group

In terms of Surgical Nursing final examination scores, the experimental group (*M* = 81.81, *SD* = 6.894) scored significantly higher than the control group (*M* = 76.63, *SD* = 9.224, *p* = 0.013) after the course, and had a moderate effect size (d = 0.636).

### Result analysis of students’ autonomous learning competencies and its dimension scores

The results of independent sample *t*-test showed that there were no significant differences in the scores of autonomous learning competencies and its self-management, information management and learning cooperation domains between the two groups before the intervention. However, the scores of autonomous learning competencies and learning cooperation domain in the experimental group were significantly better than those in the control group after intervention (*p* < 0.05). There was a moderate effect size for the intervention on improving student’ autonomous learning competencies (d = 0.606), especially in learning cooperation dimension (d = 0.781). In addition, the paired sample *t*-test results showed that these scores were significantly improved in the experimental group after the intervention (*p* < 0.05), and had a large effect size in the total score of autonomous learning competencies (d = 0.945) and learning cooperation dimension (d = 0.956). Although the differences in the total score of autonomous learning competencies (d = 0.358) and self-management dimension (d = 0.286) of the control group before and after intervention were statistically significant (*p* < 0.05), the effect size was small (see Table 3 for details).

**Table 3:** Between-and within-group comparisons for mean scores of Autonomous Learning Competencies and its domains (M ± SD)

Independent Learning Ability	Time	Experimental group (*n* = 36)	Control group (*n* = 27)	*p*-value^a^	d
Self-management	Before	34.39 ± 3.263	34.33 ± 3.258	0.947	0.018
	After	36.53 ± 3.880	35.33 ± 3.711	0.223	0.316
	*p*-value^b^	**< 0.001**	**0.020**	——	
	d	0.597	0.286		
Information management	Before	37.69 ± 4.098	37.67 ± 3.942	0.978	0.005
	After	40.19 ± 5.518	38.56 ± 3.916	0.194	0.341
	*p*-value^b^	**0.012**	0.090	——	
	d	0.514	0.227		
Learning cooperation	Before	22.53 ± 2.833	22.07 ± 2.303	0.499	0.178
	After	25.67 ± 3.680	22.93 ± 3.327	**0.003**	0.781
	*p*-value^b^	**< 0.001**	0.074	——	
	d	0.956	0.301		
Total score	Before	94.61 ± 6.262	94.07 ± 6.610	0.743	0.084
	After	102.39 ± 9.819	96.81 ± 8.562	**0.022**	0.606
	*p*-value^b^	**< 0.001**	**0.002**	——	
	d	0.945	0.358		

Note: a: Independent-samples *t*-test; b: Paired-sample *t*-test

Abbreviations: M = mean values; SD = standard deviation; d = Cohen’s d.

### Result analysis of critical thinking disposition inventory and its dimension scores

The results in Table 4 showed that there were no significant differences in the scores of CTDI-CV and its each dimension between the two groups before intervention (*p* > 0.05). Moreover, the paired sample *t*-test results showed that these scores did not change significantly in the control group after the course (*p* > 0.05), whereas the total score of CTDI-CV and scores in the domains of open-mindedness, analysis ability, inquisitiveness and cognitive maturity in the experimental group were significantly higher than before (*p* < 0.05). And the new teaching mode had a large effect size on open-mindedness dimension (d = 0.832) of the students in the experimental group, and a moderate effect size on the dimension of analysis ability (d = 0.605) and inquisitiveness (d = 0.553). Besides, the independent sample *t*-test showed that the scores of critical thinking ability (d = 0.528) and its analysis ability (d = 0.676) and inquisitiveness (d = 0.523) in the experimental group were significantly greater than those in the control group after intervention (*p* < 0.05), with moderate effect sizes (see Table 4 for details).

**Table 4:** Between-and within-group comparisons for mean scores of CTDI-CV and its domains (M ± SD)

CTDI-CV	Time	Experimental group (*n* = 36)	Control group (*n* = 27)	*p*-value^a^	d
Truth-seeking	Before	34.25 ± 9.053	34.74 ± 8.632	0.829	0.055
	After	37.31 ± 6.882	35.11 ± 4.870	0.163	0.369
	*p*-value^b^	0.063	0.773	——	
	d	0.381	0.053		
Open-mindedness	Before	35.39 ± 6.207	35.85 ± 4.769	0.748	0.083
	After	41.17 ± 7.618	38.81 ± 6.873	0.211	0.325
	*p*-value^b^	**< 0.001**	0.112	——	
	d	0.832	0.500		
Analysis ability	Before	39.56 ± 5.997	38.67 ± 5.385	0.546	0.156
	After	43.06 ± 5.570	39.30 ± 5.553	**0.010**	0.676
	*p*-value^b^	**0.008**	0.704	——	
	d	0.605	0.115		
Systematization ability	Before	36.72 ± 6.041	35.07 ± 2.947	0.160	0.347
	After	39.44 ± 7.145	36.85 ± 4.982	0.095	0.421
	*p*-value^b^	0.079	0.151	——	
	d	0.411	0.435		
Self-confidence	Before	38.33 ± 8.135	39.81 ± 6.962	0.450	0.195
	After	41.61 ± 6.834	40.04 ± 6.009	0.345	0.244
	*p*-value^b^	0.076	0.902	——	
	d	0.437	0.035		
Inquisitiveness	Before	40.50 ± 6.425	40.56 ± 6.047	0.972	0.010
	After	44.08 ± 6.522	40.89 ± 5.646	**0.046**	0.523
	*p*-value^b^	**0.004**	0.828	——	
	d	0.553	0.056		
Cognitive maturity	Before	35.78 ± 9.335	34.48 ± 9.870	0.596	0.135
	After	38.86 ± 8.043	38.63 ± 10.892	0.923	0.024
	*p*-value^b^	**< 0.001**	0.164	——	
	d	0.353	0.399		
Total score	Before	260.53 ± 34.006	259.19 ± 17.196	0.839	0.050
	After	285.53 ± 33.752	269.63 ± 25.957	**0.046**	0.528
	*p*-value^b^	**< 0.001**	0.091	——	
	d	0.738	0.474		

Note: a: Independent-samples *t*-test; b: Paired-sample *t*-test

Abbreviations: M = mean values; SD = standard deviation; d = Cohen’s d;

CTDI-CV = The Critical Thinking Disposition Inventory-Chinese Version.

### Qualitative data results

By summarizing the information provided by the interview data, five themes were extracted: (1) stimulating learning interest; (2) improving autonomous learning ability; (3) improving the sense of teamwork; (4) exercising critical thinking; and (5) suggestions for improvement in using this teaching model.

### Theme 1: stimulating learning interest

Compared to traditional teaching, BOPPPS combined with TBL teaching used abundant teaching resources (including comprehensive cases, online micro-videos, etc.) to catch students’ attention. Moreover, the application of BOPPPS combined with TBL increased the interaction between teachers and students helped stimulate students’ interest in learning.

“The comprehensive case presented to us by teachers could closely relate current events to professional knowledge, so that we can better combine theory with practice, which motivated me to continue to learn.”(S1).

“Teachers usually gave website links to each chapter before class, and we could learn directly by clicking on the links, which was faster and more convenient than blindly searching for knowledge. In addition, those small videos were very interesting and inspired my interest to learn.”(S2).

### Theme 2: improving autonomous learning ability

Teachers assigned relevant tasks before class, so that students could clarify the learning objectives of the course, and complete the pre-class test by themselves. After class, students could summarize the learning content and complete the quiz after class. This closed-loop process provided conditions for nursing students to learn independently, which was helpful to improve their autonomous learning ability.

“The personal quizzes and group assignments before class urged me to take the initiative to preview and find out useful knowledge, and the supplement of post-class tests made learning more purposeful and planned, which inspired me to learn the content of this course.”(S3).

“This kind of teaching mode made me feel more involved in the class. I become more active to learn and find solutions by myself first. When I participated in group learning, my self-esteem and sense of accomplishment would push me to study further and more proactively acquire the professional knowledge.”(S4).

“I would complete the relevant pre-class preview tasks consciously, lest I couldn’t keep up with the learning progress of teachers and groups in class.”(S5).

### Theme 3: improving the sense of teamwork

Students participated in class interaction within groups and worked together to complete team tasks (such as group tests and case discussions), which was helpful to cultivate their teamwork consciousness and ability.

“With the supervision of group cooperation, I previewed learning contents more actively before the class. I would try my best to digest and understand the learning content and search for materials according to the tasks assigned by the teacher, because I did not want to lag behind others.”(S6).

“Teachers assigned tasks for us to discuss together. Sometimes when our group had discussed a nearly perfect answer, we were often surprised at the answers of other groups. The teaching mode made me deeply realize that the strength of the team was very powerful.”(S7).

### Theme 4: exercising critical thinking

Students participated in the class in various forms, such as scenario mode, case discussion and so on. Everyone had the opportunity to express their opinions. The collision of different opinions triggered students to constantly reflect and analyze, and thus their critical thinking ability could be exercised.

“When we simulated a real case, I found that there is a certain gap between clinic and practice, and then I thought about which one is right? Is it reasonable for me to do so?”(S8).

“When the results of our discussion were different, I would not blindly listen to them, but proved right or wrong by searching for information. Teachers also encouraged us to seek evidence through literature.”(S9).

### Theme 5: suggestions for improvement

In view of the application of BOPPPS combined with TBL teaching in Surgical Nursing, students actively put forward some suggestions.

“The way of preview before class and review after class was helpful to improve my learning effect, but sometimes it took a lot of time and brought a little heavy burden. I hope teachers could upload more concise materials and exercises.”(S10).

“Some contents have been given in the pre-class preview materials, and are relatively easy to understand. Thus, there is no need to spend too much time on these contents in class.”(S11).

## Discussion

This study implemented the teaching of BOPPPS combined with TBL in the Surgical Nursing course and investigated its effects on nursing undergraduates’ learning outcomes. This new teaching approach helped to foster nursing students’ mastery of professional knowledge, and cultivate their autonomous learning competencies as well as critical thinking abilities.

### Improvements in students’ mastery of knowledge

Nursing students receiving the new teaching approach of BOPPPS and TBL obtained higher examination results than those who exposed to traditional methods. This suggested that this teaching mode of BOPPPS combined with TBL promoted their mastery of knowledge. To deal with the problem that human attention can only last about 15 min [26], BOPPPS effectively divides the learning process into six sections (including Bridge-in, Objectives, Pretest, Participatory Learning, Post-test and Summary), each of which mobilizes students’ attention in an active learning context [11, 15]. Specifically, lively and interesting introduction can capture students’ enthusiasm for the class, and clear multi-dimensional learning objectives can help students establish complete and efficient learning ideas. In our study, the individual readiness assessment test stimulates students to independently explore new knowledge and realize their weaknesses, which may be helpful focus themselves in the class. In the most important participatory learning part, team readiness assessment test inspired students to enter the group collaborative inquiry learning, and subsequent situational simulation, role-playing, reporting or presentation improved students’ classroom participation and gave full play to their respective advantages [18]. Active participation in learning helps students internalize and absorb knowledge. We found that this BOPPPS combined with TBL learning mode can help students transform passive knowledge acquisition into active knowledge seeking, which is helpful for the absorption and mastery of knowledge. At the same time, group members can deal with learning difficulties together and urge each other to study [27]. As a result, all the students in the experimental group passed the exam of the Surgical Nursing course and achieved significant improvements.

### Improvements in students’ autonomous learning competencies

Autonomous learning competency promotes nursing students to acquire nursing professional knowledge, nursing skills and ability (i.e., self-management ability, information management ability and learning cooperation ability) necessary for nursing practice [28]. Good autonomous learning competency can help nursing students to be flexible and open to adjust themselves to overcome challenges, which is an essential quality needed to develop a successful nursing career [29]. Previous studies showed that effective learning methods could effectively improve teaching quality and promote nursing students’ self-directed learning [30]. Our findings suggested that BOPPPS combined with TBL teaching could improve nursing students’ independent learning ability, especially in the areas of learning cooperation.

This study used a combination of BOPPPS with TBL creating a relaxed, interesting and cooperative learning atmosphere for students to independently study at first, and cooperatively deal with difficulties in learning process, which stimulated their subjective consciousness [31, 32] and strengthened their communication and cooperation ability [33]. The Surgical Nursing curriculum based on the BOPPPS model with six steps had clear learning objectives, prominent emphasis, and enabled students to self-monitor and made timely evaluation, which facilitated students’ self-management ability. Besides, students could arrange pre- and after-class learning tasks at their own pace, which also provided them with the opportunities for self-management [34]. In our research, the learning objectives and pre-test were set up in advance, which guided students to study deeply through access to materials, independent thinking and group discussion. This made nursing students consciously broaden their information channels, acquire knowledge and do a good job in information management before class.

The BOPPPS combined with TBL teaching in the Surgical Nursing course not only mobilized the interaction between teachers and students, but also enhanced the cooperation and communication between students and improved the ability of teamwork. Nevertheless, the differences in self-management and information management dimensions between the experimental and control groups were not significant, suggesting that there were some improvements needing to be made in the curriculum design, such as appropriately assigning the occasionally heavy pre-class preview tasks to avoid students’ tiredness of self-study.

### Improvements of students’ critical thinking disposition

Critical thinking is a judgment process involving purpose and self-supervised reflection [35], listed as one of the core abilities of nurses to effectively solve problems in complex nursing practice. Our research results showed that this BOPPPS combined with TBL model improved the critical thinking ability of nursing students, especially in the aspects of analysis ability and inquisitiveness.

The participatory learning of BOPPPS integrated learning methods such as case analysis and scenario simulation, which was highly targeted. In doing these, nursing students could be exposed to more real clinical situations during theoretical learning [11]. At the same time, TBL enabled team members to discuss together and express their own opinions, and finally draw and defend their conclusions in the participatory learning process [36, 37]. This process effectively exercised students’ analytical ability. In addition, aiming at the given cases, nursing students conducted classroom discussions, which compelled them to think and analyze the problems. When students explored clinical complex cases, their curiosity for knowledge could be arouse, which also motivated team members gather to discuss and drew on team strength to find solutions. Studying together could inspire students to think from multiple perspectives and consciously embrace multiple solutions in their search for answers [38, 39].

The BOPPPS combined with TBL teaching focused on “student-oriented” [11], enabled students to take the initiative to learn and establish their own knowledge system, and helped improve their critical thinking skills.

### Limitations and future directions

This study has some limitations. Firstly, this study was only conducted at a university in southern China with a small sample size. Therefore, the cultural context and the limited generalizability of our results should be considered. Additionally, the educational innovation described here was only conducted in a course for one semester, and thus the advantages and the longitudinal effects of BOPPPS combined with TBL learning could not be fully investigated. Thirdly, the quasi-experimental design of our study could not avoid all factors (i.e., the psychological factors of the participants). For example, voluntary participation might increase the possibility that students showed interest in nursing from the beginning joined the experiment, and thus must be taken into consideration when interpreting the results. Therefore, rigorous randomized controlled trials with large sample size are need to confirm our results and further explore the long-term effects of BOPPPS combined with TBL learning.

## Conclusions

In this study, we carried out the combined teaching mode of BOPPPS with TBL in the Surgical Nursing course. This combination not only meets students’ individualized learning needs, but also promotes cooperation and interaction among them. Our results proved that this BOPPPS combined with TBL teaching mode promoted nursing students’ autonomous learning competencies and critical thinking ability, and was feasible in the course of Surgical Nursing. In the future, the effects of this teaching model need be further probed.

## Electronic supplementary material

Below is the link to the electronic supplementary material.

Supplementary Material 1

## Data Availability

The datasets used during the current study are available from the corresponding author on reasonable request.
