# Supplementary material for: Effects of BOPPPS combined with TBL in surgical nursing for nursing undergraduates: a mixed-method study
Source: BMC Nurs. 2023 Apr 23;22:133. doi: 10.1186/s12912-023-01281-1 (PMC10122814; doi:10.1186/s12912-023-01281-1)
Supplement: Supplementary file 1 — Supplementary Material 1 [file 12912_2023_1281_MOESM1_ESM.docx]

**Appendix A: The application of BOPPPS model in different disciplines**

| **Ref.** | **Participants and Discipline** | **Teaching model** | **Implementation process of teaching in the experimental group** |
| --- | --- | --- | --- |
| [1] | Students of clinical medicine  (Experimental group 44, Control group 44);  Thoracic surgery | Experimental group: BOPPPS  Control group: Traditional teaching method | One week before the internship, students were told the theoretical chapters and related issues. The BOPPPS model was divided into six stages, and in the “participatory learning”, students were divided into groups for case discussion. |
| [2] | Paediatric undergraduates  (Experimental group 44, Control group 43);  Ophthalmology | Experimental group: BOPPPS-CBL  Control group: Traditional teaching method | Before class, cases online teaching platforms was used to introduce the main learning content, and students were asked to complete the pre-evaluation online. In class, case teaching was adopted to complete participatory learning. After class, the teacher encouraged students to study extensively. |
| [3] | Students of clinical medicine;  Physiology | Experimental group: a hybrid BOPPPS teaching model  Control group: BOPPPS | Before the lecture, teachers published the course guidance online. In the lecture, teachers used a combination of online and offline methods to introduce the course. Group discussion was used in the “participatory learning” to analyze clinical cases. After the lecture, the teacher posted a mind map online to guide students to summarize the course content. |
| [4] | 5th-year undergraduates;  Oral histopathology | BOPPPS-based SPOC and Flipped class | A proportion of students learned a knowledge unit in advance under the guidance of the teacher through the Small Private Online Course (SPOC). The knowledge gained was then presented in an online class by a representative who described the students as a “Flip class”. This process replaced the pre-assessment in the original BOPPPS. |
| [5] | The health management students  (Experimental group 55, Control group 54);  Course of Health Services Management | Experimental group: Blended learning combined with BOPPPS model  Control group: Traditional teaching method | Before class, the instructor posted cases or short videos online as the first phase of “bridge in”. In class, the instructor explained the materials through PowerPoint presentations and guided students in performing the participatory learning activities. Finally, the students and instructor summarized knowledge together. After the classroom, students were required to complete their study notes. |
| [6] | Fourth-year predoctoral dental students  (Experimental group 54, Control group 51);  Dental Materials Science | Experimental group: Microteaching Combined with the BOPPPS Model  Control group: Traditional teaching method | Microteaching is a training system to improve teaching skills, which was used in teacher training before the experiment. Then, BOPPPS was used to design the teaching process in class. |

1. Hu K, Ma RJ, Ma C, Zheng QK, Sun ZG: **Comparison of the BOPPPS model and traditional instructional approaches in thoracic surgery education**. *BMC Med Educ* 2022, **22**(1):447.

2. Chen L, Tang XJ, Chen XK, Ke N, Liu Q: **Effect of the BOPPPS model combined with case-based learning versus lecture-based learning on ophthalmology education for five-year paediatric undergraduates in Southwest China**. *BMC Med Educ* 2022, **22**(1):437.

3. Liu XY, Lu C, Zhu H, Wang X, Jia S, Zhang Y, Wen H, Wang YF: **Assessment of the effectiveness of BOPPPS-based hybrid teaching model in physiology education**. *BMC Med Educ* 2022, **22**(1):217.

4. Wang S, Xu X, Li F, Fan H, Zhao E, Bai J: **Effects of modified BOPPPS-based SPOC and Flipped class on 5th-year undergraduate oral histopathology learning in China during COVID-19**. *BMC Med Educ* 2021, **21**(1):540.

5. Ma X, Ma X, Li L, Luo X, Zhang H, Liu Y: **Effect of blended learning with BOPPPS model on Chinese student outcomes and perceptions in an introduction course of health services management**. *Adv Physiol Educ* 2021, **45**(2):409-417.

6. Yang Y, You J, Wu J, Hu C, Shao L: **The Effect of Microteaching Combined with the BOPPPS Model on Dental Materials Education for Predoctoral Dental Students**. *J Dent Educ* 2019, **83**(5):567-574.
